# TAT-Conjugated NDUFS8 Can Be Transduced into Mitochondria in a Membrane-Potential-Independent Manner and Rescue Complex I Deficiency

**DOI:** 10.3390/ijms22126524

**Published:** 2021-06-17

**Authors:** Bo-Yu Lin, Gui-Teng Zheng, Kai-Wen Teng, Juan-Yu Chang, Chao-Chang Lee, Pin-Chao Liao, Mou-Chieh Kao

**Affiliations:** 1 Institute of Molecular Medicine, National Tsing Hua University, Hsinchu 30013, Taiwan; bobo5230@hotmail.com (B.-Y.L.); ing597859781@gmail.com (G.-T.Z.); lin66556@gmail.com (K.-W.T.); juanyu0124@hotmail.com (J.-Y.C.); kirbylee0921@gmail.tw (C.-C.L.); packliao@gmail.com (P.-C.L.); 2 Department of Life Science, College of Life Science, National Tsing Hua University, Hsinchu 30013, Taiwan

**Keywords:** mitochondria, complex I, NDUFS8, protein transduction domain, HIV-1 transactivator of transcription peptide (TAT), enzyme replacement therapy, mitochondrial targeting sequence, mitochondrial membrane potential

## Abstract

NADH dehydrogenase (ubiquinone) Fe-S protein 8 (NDUFS8) is a nuclear-encoded core subunit of human mitochondrial complex I. Defects in NDUFS8 are associated with Leigh syndrome and encephalomyopathy. Cell-penetrating peptide derived from the HIV-1 transactivator of transcription protein (TAT) has been successfully applied as a carrier to bring fusion proteins into cells without compromising the biological function of the cargoes. In this study, we developed a TAT-mediated protein transduction system to rescue complex I deficiency caused by NDUFS8 defects. Two fusion proteins (TAT-NDUFS8 and NDUFS8-TAT) were exogenously expressed and purified from *Escherichia coli* for transduction of human cells. In addition, similar constructs were generated and used in transfection studies for comparison. The results showed that both exogenous TAT-NDUFS8 and NDUFS8-TAT were delivered into mitochondria and correctly processed. Interestingly, the mitochondrial import of TAT-containing NDUFS8 was independent of mitochondrial membrane potential. Treatment with TAT-NDUFS8 not only significantly improved the assembly of complex I in an NDUFS8-deficient cell line, but also partially rescued complex I functions both in the in-gel activity assay and the oxygen consumption assay. Our current findings suggest the considerable potential of applying the TAT-mediated protein transduction system for treatment of complex I deficiency.

## 1. Introduction

Human complex I, also called NADH-coenzyme Q oxidoreductase, is the first, largest, and most complicated respiratory complex in the oxidative phosphorylation system (OXPHOS), which is not only critical in catalyzing NADH oxidation to transfer two electrons to ubiquinone but also in coupling proton translocation for ATP generation [1,2]. In addition, it is the major source of reactive oxygen species (ROS) production [2,3,4]. This protein complex has an L-shaped structure containing a peripheral arm protruding in the matrix and a membrane arm embedded in the inner membrane of mitochondria [5]. It is composed of 45 (44 unique) subunits with a molecular size of about 1000 kDa [5,6,7]. Among them, 7 subunits (ND1–6 and ND4L) are encoded by mitochondrial DNA (mtDNA) and 37 subunits (NDUFV1–3, NDUFS1–8, NDUFA1–13, NDUFB1–11, NDUFAB1, and NDUFC1–2) are encoded by nuclear DNA (nDNA) [1,8]. Due to its dual origins, structural complexity, and important roles in OXPHOS, complex I deficiency has been linked to a large variety of pathological conditions such as fatal infantile lactic acidosis (FILA), Leigh syndrome [9,10], Leber hereditary optic neuropathy (LHON), and mitochondrial encephalomyopathy with lactic acidosis (MELAS) [8], and is associated with the pathogenesis of many devastating neurodegenerative disorders including Parkinson’s disease [11,12].

NADH dehydrogenase (ubiquinone) Fe-S protein 8 (NDUFS8) is a nuclear-encoded subunit of human complex I. This protein and the other 13 proteins (NDUFS1–3, NDUFS7, NDUFV1–2, ND1–6, and ND4L) are considered the core subunits of complex I, which are highly conserved from prokaryotic to eukaryotic cells. NDUFS8 contains two tetranuclear iron-sulfur clusters (N6a and N6b) and plays a key role in electron transfer in complex I. NDUFS8 deficiency caused by missense mutations in *NDUFS8* gene was found as the first molecular genetic link between a nuclear-encoded subunit of complex I and Leigh syndrome, which is a severe neurological disorder characterized by bilaterally symmetrical necrotic lesions in the subcortical areas of the brain [13]. In addition, a novel mutation in this gene was found in children with encephalomyopathy [14]. The discoveries from recent studies also indicated that *NDUFS8* gene is one of eight OXPHOS component genes that are downregulated in patients with bipolar disorder (BD) [15].

Defects directly or indirectly affecting the function of OXPHOS are the most frequent causes of mitochondrial diseases. However, due to its dual origins, the complexity of the system, and the diversity of clinical phenotypes, reliable and convenient therapeutic approaches for the treatment of disorders caused by OXPHOS deficiency are still lacking. Most conventional treatments currently available for mitochondrial diseases are only palliative. Enzyme replacement therapy (ERT) [16], a therapeutic approach for replacing a defective enzyme in patients with a normal one, has recently emerged as a promising approach to treating mitochondria-related disorders. However, to successfully apply this approach in rescuing OXPHOS deficiency, the barrier imposed by plasma membranes that separate the interior of cells from the surrounding environment and thus block the transduction of therapeutic proteins has to be conquered. This limitation can be surmounted by the conjugation of a protein transduction domain ((PTD) also called cell-penetrating peptide (CPP)), which is a short peptide (less than 30 amino acids) capable of crossing cell membranes with no cell-type specificity and thus facilitates the delivery of protein cargoes into cells [17].

Since the discovery of the cell-penetrating function of PTDs, many PTDs have been identified and designed, including TAT (from human immunodeficiency virus (HIV)-transactivator of transcription protein) [18,19], Antp (from *Drosophila* homeotic transcription protein antennapedia) [20], VP22 (from *Herpes simplex* virus 1 DNA-binding protein VP22) [21], and polyarginines (chemically synthesized) [22]. Among them, TAT peptide and its derivatives are the most adopted and relatively well-characterized. The cationic charges of this peptide were shown to play an essential role in the cellular uptake process, and the minimal sequence with protein transduction function is the amino acid residues 49–57 (RKKRRQRRR) of the original TAT protein [23]. To deliver cargo proteins to certain organelles with PTDs, the presence of a specific organelle signaling peptide in the PTD-containing fusion proteins is required for proper targeting [24]. Mitochondrial targeting sequence (MTS) is an N-terminal cleavable signal peptide that can assist with the mitochondrial targeting and entrance of about 60–70% of nuclear-encoded mitochondrial precursor proteins through a presequence import pathway toward the mitochondrial matrix [25,26,27]. This canonical mitochondrial import pathway is driven by both the mitochondrial membrane potential (ΔΨm) and ATP generated via OXPHOS.

Del Gaizo et al. published the first report demonstrating that TAT incorporated in front of an MTS from mitochondrial malate dehydrogenase (mMDH) can achieve plasma membrane crossing and mitochondrial delivery and accumulation of an exogenous enhanced green fluorescent protein (eGFP) protein [28,29]. However, while attempting to apply the same concept by the addition of an MTS to exonuclease III (ExoIII) to accomplish the repair of mtDNA damage to the mitochondria of breast cancer cells following oxidative stress, Shokolenko et al. found that the successful delivery of TAT-fused ExoIII into mitochondria occurred only when an MTS was present as the first domain in the N-terminus of the recombinant protein as an MTS–ExoIII–TAT fusion [30]. The above finding implicated that the presence of TAT preceding an MTS might hamper the mitochondrial targeting of the fusion protein. Nevertheless, Rapoport et al. later succeeded in applying TAT conjugation in front of a nuclear-encoded mitochondrial lipoamide dehydrogenase (TAT–LAD) to deliver the fusion protein into mitochondria to restore pyruvate dehydrogenase complex activity in the mitochondria of patients with LAD deficiency [31], and in multiple tissues in the LAD-deficiency model mice [32].

According to the results of the aforementioned studies, the mechanism of delivering proteins to mitochondria by incorporation of TAT with an MTS still remains controversial and elusive. Due to the essential roles of NDUFS8 in complex I function and structure, and the importance of complex I in OXPHOS, we tried to apply the concept of ERT with the combination of the membrane crossing ability of TAT and the mitochondrial targeting capability of MTS in NDUFS8 to develop a novel TAT-mediated protein transduction system to deliver exogenously produced NDUFS8 into mitochondria in this study. The results indicated that, with their native MTS, both exogenously produced TAT-NDUFS8 and NDUFS8-TAT were delivered into mitochondria and processed into the mature forms of NDUFS8. Surprisingly, this delivery system was independent of ΔΨm, which is dramatically different from the result of another endogenously expressed, nuclear-encoded complex I component protein NDUFS3 [1,8], which was used for comparison of ΔΨm dependency on crossing the mitochondrial inner membrane in this study. Furthermore, treatment with TAT-NDUFS8 fusion protein partially restored the assembly and functionality of complex I in NDUFS8-deficient cells. Our findings demonstrate the potential application of this protein transduction system for therapeutic treatment of complex I deficiency caused by NDUFS8 defects.

## 2. Results

### 2.1. The Fusion Protein Derived from Transient Transfection of TAT-NDUFS8 Could Not Correctly Enter Mitochondria 

**Figure 1:**
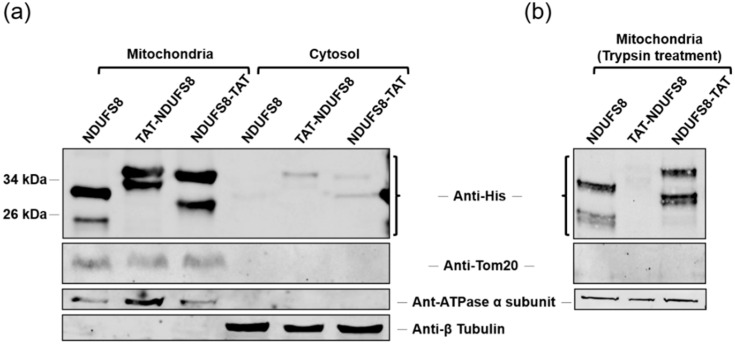
The fusion protein derived from transient transfection of TAT-NDUFS8 could not correctly enter mitochondria. T-REx-293 cells were transiently transfected with TAT-NDUFS8, NDUFS8-TAT, or NDUFS8 plasmid for 18 h. After subcellular fractionation, the mitochondrial fractions were untreated (**a**) or treated with trypsin (**b**), followed by immunoblotting analysis. The His-tagged fusion proteins were detected with anti-His antibody. The protein markers of the obtained fractions were detected with specific antibodies to verify the correct subcellular fractionation and used as the loading controls: β-tubulin was used as a cytosolic marker; ATPase α subunit was used as a mitochondrial inner membrane marker; Tom20 was used as an outer mitochondrial membrane marker.

To explore the contradictory findings regarding the relative location of TAT to an MTS and the cargo protein in the delivery of a PTD-containing protein to mitochondria, three plasmids, each with a TAT-NDUFS8, NDUFS8-TAT, or NDUFS8 insert carrying a His tag for identification, were constructed. The primers used are listed in Table 1. Transient transfection with each of the aforementioned NDUFS8-containing constructs was first conducted on T-REx-293 cells, followed by subcellular fractionation and immunoblotting analysis to detect the localization of target proteins. Theoretically, the MTS of the nuclear-encoded mitochondrial NDUFS8 would target the protein to mitochondria and then be cleaved by mitochondrial processing peptides (MPPs) [25,26,27]. As a positive control, two bands, including the precursor and mature forms of recombinant NDUFS8, were detected by anti-His antibody in the mitochondrial fraction with a size of about 30 and 25 kDa, respectively (Figure 1a). As expected, NDUFS8-TAT, whose MTS is not preceded by the TAT addition, showed a similar pattern to that of NDUFS8 without TAT tagging (both the precursor and mature forms were observed). Interestingly, two bands were also detected in the mitochondrial fraction of cells transfected with TAT-NDUFS8. However, considering their corresponding sizes, the upper band was expected to be the precursor form of TAT-NDUFS8 but the lower band could be a degraded product, not the mature form of this fusion protein in the mitochondria. These findings suggested that the addition of TAT in the N-terminus of a nuclear-encoded mitochondrial protein may shelter its MTS and thus prevent the correct processing of the fusion protein TAT-NDUFS8 in mitochondria. To determine whether the detection of TAT-NDUFS8 precursor in the mitochondrial fraction was due to the protein attached to the mitochondrial outer membrane, trypsin was used to digest the outer-membrane-associated proteins. The results revealed that the outer membrane marker Tom20 disappeared after trypsin treatment (Figure 1b). Conversely, the inner membrane marker ATPase α subunit was not affected by treatment with trypsin. Importantly, TAT-NDUFS8 was undetectable after the treatment with trypsin. This observation indicated that the TAT-NDUFS8 expressed by transient transfection was proteolyzed by trypsin since it cannot translocate correctly into mitochondria but sticks on the outer mitochondrial membrane. These findings showed that only NDUFS8-TAT but not TAT-NDUFS8 can be correctly delivered to mitochondria by transient transfection.

### 2.2. TAT Conjugation Was Required for Protein Transduction of Exogenously Produced NDUFS8 into T-REx-293 Cells

**Figure 2:**
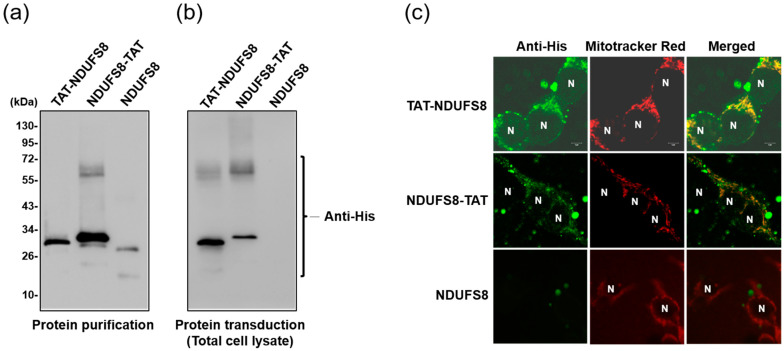
TAT conjugation was required for protein transduction of exogenously produced NDUFS8 into T-REx-293 cells. (**a**) TAT-NDUFS8, NDUFS8-TAT, and NDUFS8 recombinant proteins were overexpressed in *E. coli* and purified by Co^2+^ affinity chromatography. Immunoblotting analysis was conducted on the purified recombinant proteins with anti-His antibody. The effectiveness of TAT conjugation on the delivery of exogenously produced NDUFS8 into cells through protein transduction for 3 h was evaluated by immunoblotting analysis on the total cell lysates with anti-His antibody (**b**), and by immunofluorescence detection with the confocal microscopy on the whole cells (**c**). For immunofluorescence analysis, anti-His primary antibody together with anti-IgG-FITC secondary antibody (green staining) was used to detect NDUFS8-containing recombinant proteins, and Mitotracker Red signal (red staining) was applied to locate mitochondria. N, the nucleus. Scale bar = 5 μm.

TAT-NDUFS8, NDUFS8-TAT, and NDUFS8 recombinant proteins used for later protein transduction studies were exogenously overexpressed in *E. coli* BL21 (DE3) and purified by Co^2+^ affinity chromatography. Protein samples collected from 250 and 500 mM imidazole elution were added together and used for buffer exchange with phosphate-buffered saline (PBS). These purified proteins were then detected by immunoblotting analysis with anti-His antibody. As shown in Figure 2a, although some degraded and aggregated forms of products were observed, all three NDUFS8-containing recombinant proteins appeared as a dominant band with the expected molecular size, suggesting that protein overexpression and purification all succeeded in these three versions of exogenously expressed NDUFS8 proteins.

To investigate the effectiveness of TAT addition on facilitating the delivery of exogenously produced NDUFS8 protein cargo into cells, protein transduction experiments were performed. T-REx-293 cells were incubated with TAT-NDUFS8, NDUFS8-TAT, or NDUFS8, followed by immunoblotting analysis with anti-His antibody on the total cell lysate samples. As shown in Figure 2b, after protein transduction treatment, both TAT-NDUFS8 and NDUFS8-TAT were present in the total cell lysates. In contrast, NDUFS8 was absent from the total cell lysates. To confirm the above finding, the result of TAT-facilitating cell entry of NDUFS8 was verified by immunofluorescence detection using the confocal microscopy approach. The resulting images showed that most TAT-NDUFS8 and NDUFS8-TAT l found to be within T-REx-293 cells and colocalized with mitochondria (labeled using Mitotracker Red treatment), whereas no NDUFS8 signal was observed in cells treated with NDUFS8 (Figure 2c). These observations suggested that TAT conjugation is a necessity for protein transduction of exogenously produced NDUFS8 into T-REx-293 cells.

### 2.3. Transduction of Exogenously Produced TAT-NDUFS8 and NDUFS8-TAT across the Mitochondrial Inner Membrane Was Independent of ΔΨm

**Figure 3:**
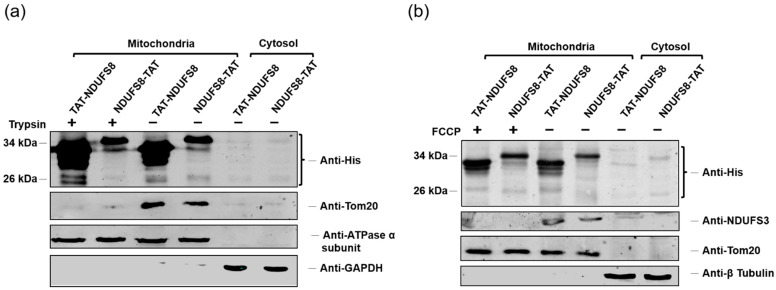
Transduction of exogenously produced TAT-NDUFS8 and NDUFS8-TAT across the mitochondrial inner membrane was independent of ΔΨm. (**a**) The effects of trypsin treatment on the mitochondrial targeting and processing of TAT-NDUFS8 or NDUFS8-TAT introduced by protein transduction. After protein transduction and subcellular fractionation, the mitochondrial fractions were treated with or without trypsin. (**b**) The effects of FCCP treatment on the protein translocation of TAT-NDUFS8 or NDUFS8-TAT across the mitochondrial inner membrane. Cells were cultivated with or without FCCP for 18 h, followed by protein transduction with TAT-NDUFS8 or NDUFS8-TAT for another 3 h. (**a**,**b**) After subcellular fractionation, immunoblotting analysis was performed. The His-tagged fusion proteins were detected with anti-His antibody. The protein markers of the obtained fractions were detected with specific antibodies to verify the correct subcellular fractionation and used as the loading controls: β-tubulin and GAPDH were used as cytosolic markers; ATPase α subunit was used as an inner mitochondrial membrane marker; Tom20 was used as an outer mitochondrial membrane marker; NDUFS3 was used as a mitochondrial matrix marker.

The results of the previous section indicated that both recombinant TAT-NDUFS8 and NDUFS8-TAT can enter into T-REx-293 cells and target to mitochondria through the process of protein transduction. To further confirm the mitochondrial targeting and exclude the possibility that these two TAT-containing NDUFS8 proteins might be just associated with the outer mitochondrial membrane but not truly translocated into mitochondria, T-REx-293 cells incubated with each of these TAT fusion proteins were analyzed by subcellular fractionation, followed with or without trypsin treatment and immunoblotting analysis. The results shown in Figure 3a demonstrate that both TAT-NDUFS8 and NDUFS8-TAT were present in the mitochondrial fraction; in addition to the original precursor proteins, a smaller protein (sometimes appeared as a doublet) with a molecular size corresponding to the mature form of fusion protein was detected not only for NDUFS8-TAT, but also for TAT-NDUFS8 after protein transduction treatment. Moreover, the presence of two forms of TAT-NDUFS8 and NDUFS8-TAT in the mitochondrial fraction was not significantly affected by trypsin treatment. These findings suggested both exogenously produced TAT-NDUFS8 and NDUFS8-TAT can enter mitochondria and be successfully processed into the mature form of fusion protein. Compared to the results of our previous study on the transient transfection of TAT fusion proteins, the current findings imply that there might be a distinct protein import pathway for the delivery of exogenous TAT-containing NDUFS8 into mitochondria by protein transduction.

In typical mitochondrial import pathways, it is well-documented that ΔΨm is required for driving proteins across the mitochondrial inner membrane to reach their final destiny in the matrix or the inner mitochondrial membrane [25,26,27]. To verify whether a different mitochondrial import pathway exists for the translocation of exogenously produced TAT fusion proteins into mitochondria, carbonyl cyanid *p*-(trifluoromethoxy) phenylhydrazone (FCCP) was added in the cell culture medium to depolarize ΔΨm. As shown in Figure 3b, after the cells were treated with FCCP, the delivery and processing of TAT-NDUFS8 and NDUFS8-TAT were still be detected in the mitochondrial fraction, indicating that these two TAT fusion proteins might cross the inner mitochondrial membrane independent of ΔΨm. The signal detected by anti-NDUFS3 antibody for an endogenous NDUFS3, another nuclear-encoded mitochondrial protein located at the matrix part of complex I [1,8], was completely lost, suggesting that the treatment with FCCP on cells affected the regular mitochondrial protein import pathway. These results support our hypothesis that the transduction of exogenous TAT-containing fusion proteins into mitochondria does not occur through the classical ΔΨm-dependent mitochondrial import pathway.

### 2.4. Overdosage of Exogenously Produced NDUFS8-TAT Was Harmful to Cell Viability

**Figure 4:**
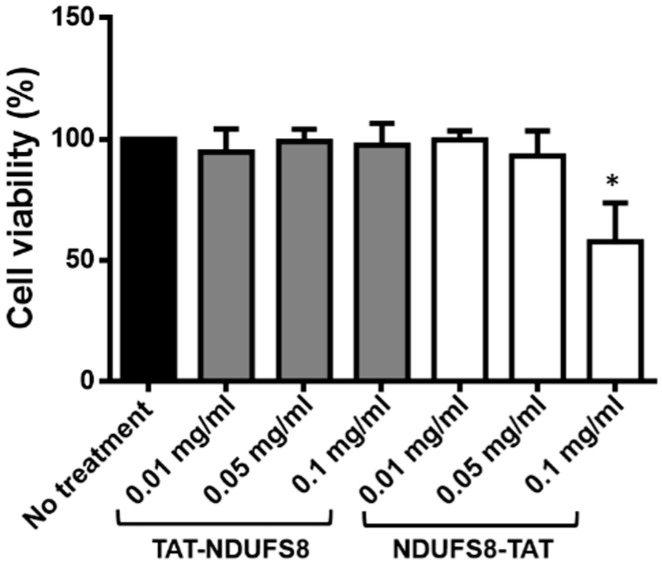
Overdosage of exogenously produced NDUFS8-TAT was harmful to cell viability. T-REx-293 cells were seeded in 96-well plates and incubated with the indicated concentration of TAT-NDUFS8 or NDUFS8-TAT for 48 h. Cell viability was measured by the MTT assay. Mean values of cell viability are shown as percentages relative to the untreated cells (*n* = 3; * *p* < 0.05).

The utility of applying a protein for the therapeutic purpose should not have undesired effects on the target cells. To evaluate the safety and dosage of applying these TAT-containing NDUFS8 proteins, the MTT (3-(4,5-cimethylthiazol-2-yl)-2,5-diphenyl tetrazolium bromide) assay was conducted. As shown in Figure 4, the cell viability decreased significantly when T-REx-293 cells were treated with 0.1 mg/mL NDUFS8-TAT. Conversely, the treatment of TAT-NDUFS8 on cells at the same concentration did not influence cell viability. Our previous results suggested that both exogenously produced TAT-NDUFS8 and NDUFS8-TAT can be delivered into mitochondria. Therefore, according to the aim of safe therapy and mitochondrial targeting, TAT-NDUFS8 is more suitable and was selected for further functional analyses in the following rescue studies on NDUFS8-deficient cells.

### 2.5. The Successful Knockdown of NDUFS8 Gene Expression by RNA Interference on shRNA-C3 Cells Was Demonstrated Both at the mRNA and Protein Levels

**Figure 5:**
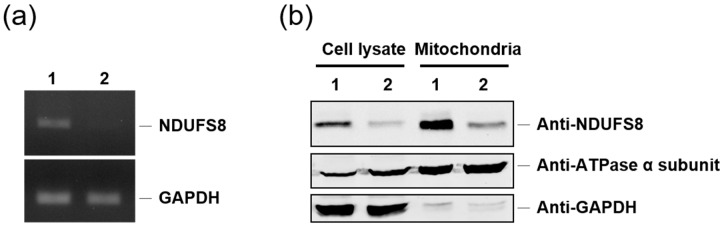
The successful knockdown of *NDUFS8* gene expression by RNA interference on shRNA-C3 cells was observed both at the mRNA and protein levels. (**a**) The *NDUFS8* expression level in T-REx293 and NDUFS8 knockdown cell line shRNA-C3 was determined by RT-PCR. (**b**) The protein level in T-Rex-293 and NDUFS8 knockdown cell line shRNA-C3 was determined on the total cell lysate and the mitochondrial fraction by immunoblotting analysis with anti-NDUFS8 antibody. GAPDH was used as an endogenous control in RT-PCR analysis and as a cytosolic marker in immunoblotting analysis; ATPase α subunit was used as a mitochondrial marker. 1, wild-type T-REx293; 2, NDUFS8 knockdown cell line shRNA-C3.

To evaluate the rescuing efficiency of applying protein transduction with the TAT-NDUFS8 fusion protein, a cell model system that can mimic the cells with NDUFS8 deficiency is needed. Recently, we generated an *NDUFS8* knockdown stable cell line (shRNA-C3) derived from T-REx-293 using the RNA interference technique to study the involvement of NDUFS8 in complex I functionality and assembly (manuscript in preparation). To verify the efficiency of NDUFS8 knockdown in shRNA-C3 cells, the mRNA level of *NDUFS8* was measured by reverse-transcription polymerase chain reaction (RT-PCR). Compared to wild-type T-REx-293, shRNA-C3 had a drastic decrease in the *NDUFS8* mRNA level (Figure 5a). In addition, the protein level of wild-type and shRNA-C3 cells was also analyzed by immunoblotting analysis of the samples from the total cell lysate and the mitochondrial fraction. As shown in Figure 5b, for both types of samples, the protein level of NDUFS8 in shRNA-C3 cells was significantly lower compared to that in the wild-type cells. Thus, we determined the shRNA-C3 cell line could be applied as an NDUFS8-deficient cell model in our later functional rescue studies.

### 2.6. Protein Transduction of Exogenously Produced TAT-NDUFS8 Improvde the Assembly of Mitochondrial Complex I and Restorde the Complex I In-Gel Activity in shRNA-C3 Cells

Based on the previous findings in this study, exogenously produced TAT-NDUFS8 can enter cells, target mitochondria, and further be processed into a mature form of NDUFS8 fusion protein. However, due to proper protein assembly also playing a critical role in the functionality of mitochondrial complex I, it was important to examine the influence of TAT-NDUFS8 fusion protein on the assembly of complex I. High-resolution clear native gel electrophoresis (HrCNE), a method derived from the original blue native polyacrylamide gel electrophoresis (BN-PAGE) [33], has gained popularity as a technique to separate intact *n*-dodecyl β-D-maltoside (DDM)-solubilized mitochondrial membrane-bound protein complexes, determine the integrity of mitochondrial respiratory chain complexes, and directly detect the enzymatic activity in gel. Here, HrCNE was applied to investigate the incorporation of TAT-NDUFS8 into complex I.

**Figure 6:**
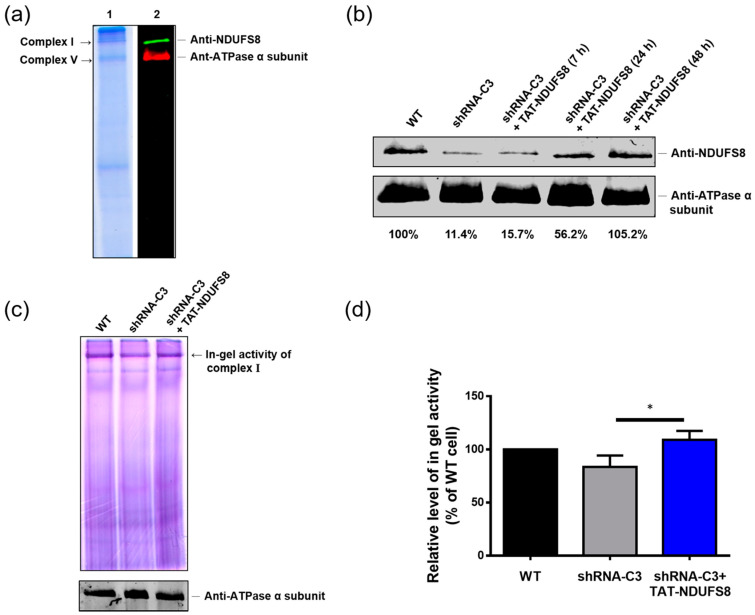
Protein transduction of exogenously produced TAT-NDUFS8 improved the assembly of mitochondrial complex I and restored the complex I in-gel activity in shRNA-C3 cells. (**a**) The protein profile of mitochondria-enriched samples derived from wild-type T-REx-293 cells. Mitochondrial membrane protein complexes solubilized by DDM from T-REx-293 cells were separated by HrCNE, and the protein profile and the location of protein complexes of the extract were visualized by Coomassie blue staining and immunoblotting analysis, respectively. (**b**) The effect of TAT-NDUFS8 treatment on the status of complex I assembly in shRNA-C3 cells. After protein transduction with 0.03 mg/mL TAT-NDUFS8 for different periods of time as indicated and subcellular fractionation, the obtained mitochondria were solubilized with DDM, resolved by HrCNE, and immunostained with anti-NDUFS8 antibody. Staining of ATPase α subunit in complex V was used as the loading control. (**c**) The effect of TAT-NDUFS8 treatment on complex I in-gel activity in shRNA-C3 cells. The obtained gel from HrCNE was incubated with NADH and NBT to perform in-gel activity staining. The amount of ATPase α subunit in complex V was detected by anti-ATPase α subunit antibody and used as a loading control. (**d**) The in-gel activity of complex I was estimated by ImageJ software, normalized to the amount of ATPase α subunit, and is shown as a percentage relative to that of T-REx-293 cells (*n* = 3; * *p* < 0.05).

By HrCNE, the protein profile of mitochondria-enriched samples derived from wild-type T-REx-293 cells is displayed in Figure 6a. Notably, the bands of 1000 kDa complex I and 550 kDa complex V were clearly recognizable in the gel not only by Coomassie blue staining, but also by immunoblotting analysis with anti-NDUFS8 and anti-ATPase α subunit antibody, respectively, after being transferred to a nitrocellulose membrane. When shRNA-C3 cells (NDUFS8-deficienct) were treated with 0.03 mg/mL TAT-NDUFS8 for different periods of time, the intensity of NDUFS8 in complex I location, which represents the degree of NDUFS8 incorporation in complex I, steadily increased from 11.4% at 0 h to 105.2% at 48 h (Figure 6b). Thus, the result demonstrated that TAT-NDUFS8 can be incorporated into complex I and increase the NDUFS8 level in complex I of shRNA-C3 cells in a time-dependent manner. Next, an in-gel activity assay was conducted to verify if the incorporation of TAT-NDUFS8 with complex I may further improve the activity of complex I. Wild-type and shRNA-C3 cells treated with or without 0.03 mg/mL TAT-NDUFS8 for 48 h were harvested, solubilized by DDM, and separated by HrCNE. Then, the obtained native gel was incubated with NADH and nitroblue tetrazolium (NBT) to perform in-gel activity staining for complex I. As shown in Figure 6c, the in-gel activity of complex I displayed a major purple-colored band near the top of the stained gel. Figure 6d shows the statistical analysis of three independent in-gel activity assays calculated with ImageJ software after normalization to the amount of ATPase α subunit displayed in the bottom panel of Figure 6c The in-gel activity of shRNA-C3 cells was about 83.6% of the wild-type activity, and the treatment of TAT-NDUFS8 significantly increased the in-gel activity of shRNA-C3 cells to 109.1% of the wild-type activity, suggesting protein transduction of TAT-NDUFS8 can substantially increase the complex I activity in shRNA-C3 cells.

### 2.7. TAT-NDUFS8 Treatment Significantly Improved the Complex I-Specific Oxygen Consumption Rate of Respiratory Chain in shRNA-C3 Cells

**Figure 7:**
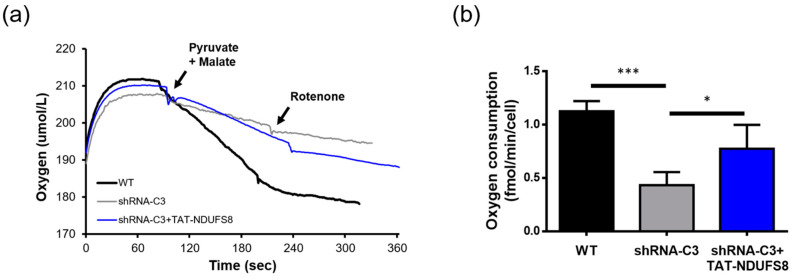
TAT-NDUFS8 treatment significantly improved the complex I-specific oxygen consumption rate of respiratory chain in shRNA-C3 cells. (**a**) The illustration of complex I-specific oxygen consumption assay of digitonin-permeabilized cells. (**b**) The oxygen consumption rate of cells was calculated from the slope of the reaction curve (*n* = 4; * *p* < 0.05; *** *p* < 0.001).

The respiratory chain of OXPHOS receives electrons from oxidized substrates and finally reduces oxygen into water. Oxygen consumption analysis can be used to evaluate the mitochondrial respiratory function. By adding complex-I-specific substrates (pyruvate and malate) and inhibitor (rotenone) during oxygen consumption analysis, the respiratory activity of complex I was measured in digitonin-permeabilized cells in this study. Figure 7a shows the oxygen concentration change during the entire experimental process. The oxygen consumption rate was calculated from the slope of the oxygen concentration curve between the substrate addition (malate and pyruvate) and the inhibitor addition (rotenone). As shown in Figure 7b, the oxygen consumption rate of shRNA-C3 cells was about 38.4% of that of the wild-type cells, and treatment with TAT-NDUFS8 significantly increased the oxygen consumption rate of shRNA-C3 cells to 68.9% of that of the wild-type cells. In total, the functional studies including the in-gel activity assay and oxygen consumption assay indicated that the treatment with exogenously produced TAT-NDUFS8 to shRNA-C3 cells increased the activity in defective complex I about 31% and 79%, respectively, suggesting that protein transduction of TAT-NDUFS8 can partially restore the activity of complex I.

## 3. Discussion

In this study, we generated a protein transduction system using TAT as the PTD to rescue the deficiency caused by the dysfunction of the NDUFS8 subunit in the mitochondrial complex I. According to our results, both exogenously produced TAT-NDUFS8 and NDUFS8-TAT could enter mitochondria; this finding was further confirmed by the results of the trypsin digestion tests. In addition, by including an uncoupler of OXPHOS, FCCP, in the experimental setting, we presented a novel mitochondrial protein import pathway in which the translocation of these two TAT fusion proteins across the inner mitochondrial membrane was independent of ΔΨm. Furthermore, using TAT-NDUFS8 as an example, we showed that after treating exogenously produced TAT-NDUFS8 for 48 h, the level of complex I assembly increased from 11.4% to 105.2% in the NDUFS8 knockdown cell line, shRNA-C3 compared to that of normal cells. Moreover, we found that the activity of complex I in shRNA-C3 cells also increased about 31% and 79% in the in-gel activity assay and the oxygen consumption assay, respectively. These data proved that this TAT-mediated NDUFS8 transduction system can partially restore the activity of complex I in shRNA-C3 cells.

Based on the data presented here, we clearly demonstrate that TAT conjugation plays a pivotal role in bringing exogenously produced NDUFS8 fusion proteins into T-REx-293 cells. When a protein transduction system is applied to deliver a TAT-containing protein into mitochondria, some contradictory findings regarding the relative location of TAT to an MTS and the cargo protein (at the N- or C-terminus) have been reported [28,29,30]. Del Gaizo et al. showed that the incorporation of TAT in front of an MTS from mMDH (MTS_mMDH_) and the target protein eGFP provides the advantage of promoting the accumulation of eGFP in mitochondria because after processing by MPPs, TAT-MTS_mMDH_ is released from the precursor fusion protein and the mature cargo eGFP is formed and retained in the mitochondria [28,29]. In contrast, when using ExoIII as the cargo protein, Shokolenko et al. found that only MTS-ExoIII-TAT fusion but not TAT-MTS-ExoIII fusion could be correctly transduced into mitochondria [30]. Importantly, the authors suggested that the presence of TAT preceding an MTS would impede the mitochondrial targeting of the fusion protein. They emphasized that the successful delivery of TAT fusion proteins into mitochondria occurs only when an MTS is present as the first domain in the N-terminus of the recombinant protein. In our current study, both TAT-NDUFS8 and NDUFS8-TAT fusion proteins were successfully transduced into mitochondria and correctly processed, indicating that the relative location between TAT and MTS (also the cargo) does not affect the mitochondrial targeting of NDUFS8. The discrepancy between ours and others’ results seem to imply that the importance of the location of TAT sequence relative to an MTS (and thus the target protein) is MTS- and/or cargo-protein-dependent. Therefore, the position of TAT and the selection of MTS have to be optimized for each individual therapeutic protein desired for targeting to mitochondria by TAT-mediated protein transduction.

Although accumulative evidence has proven that TAT-mediated delivery of proteins into cells is applicable, the mechanism through which proteins are transduced to mitochondria by incorporation with TAT still remains elusive. Some reports considered that the TAT-conjugated protein enters mitochondria via the canonical mitochondrial import pathway [30], but others suggested that the TAT delivery system may be achieved through a different mechanism that does not involve the canonical mitochondrial import pathway [29]. To clarify this unresolved mystery, we applied the transient transfection approach using TAT-derived NDUFS8 plasmids as the classic mode of the mitochondrial import pathway and compared the results to those of the TAT-mediated NDUFS8 transduction system under similar experimental conditions. Unlike the finding from the transient transfection experiment where only NDUFS8-TAT was successfully imported and processed in mitochondria, both the exogenously produced TAT-NDUFS8 and NDUFS8-TAT entered mitochondria and were then processed. In addition, when NDUFS8-TAT fusion was used for comparison, the efficiency of processing NDUFS8-TAT into its corresponding mature protein was more prominent in the transient transfection experiment than that in the protein transduction experiment. The above findings all indicate that the pathway for the mitochondrial import of TAT-conjugated NDUFS8 by protein transduction is different from the canonical mitochondrial import pathway as demonstrated by the transient transfection experiment.

Interestingly, we presented here that the translocation of exogenously produced TAT-NDUFS8 and NDUFS8-TAT across the inner mitochondrial membrane through protein transduction is independent of ΔΨm. This unexpected finding was also reported by Del Gaizo et al. when the aforementioned TAT-MTS_mMDH_-eGFP was applied in a mitochondrial targeting study [29]. The nuclear-encoded complex I protein NDUFS8 is predicated by the Mitoprot II program [34] to contain the first 34 amino acids in the N-terminus as an MTS, and the presence of two peptides corresponding to the precursor and mature forms of NDUFS8 in the results of our studies proves this prediction. For a cleavable, presequence-type mitochondrial matrix protein, NDUFS8 is expected to be imported into mitochondria through the outer mitochondrial membrane (TOM) to a classical presequence import pathway, which is involved with the presequence translocase of the inner membrane (TIM23) before being excised by MPPs. It is well-accepted that the positively charged MTS initiates electrophoretic translocation through the inner membrane, and ΔΨm is critical for translocase activation and presequence transport [25,26,27]. Therefore, the feature of ΔΨm independency of our TAT-mediated NDUFS8 transduction system suggests that it is unlike the well-recognized presequence import pathway and may be a yet-to-be-discovered route for mitochondrial matrix protein import. One example of a TIM23-dependent process of mitochondrial protein import but without ΔΨm dependency was reported in the literature [35]. The subunit e of the F_1_F_o_ ATP-synthase is a nuclear-encoded, single-spanning mitochondrial protein that is anchored to the inner membrane and lacks a recognizable MTS. It was shown that the assembly of subunit e into the inner mitochondrial membrane does not require ΔΨm. It was assumed that this ΔΨm-independence is due to the absence of positively charged amino acid residues at the N-terminus of the subunit. However, how TIM23 function is involved in the mitochondrial import of subunit e is still poorly understood. NDUFS8 possesses a cleavable N-terminal MTS with four positively charged residues that are unlike the features shown in the subunit e of the F_1_F_o_ ATP synthase. Therefore, the reasons contributing to the ΔΨm-independence of subunit e mitochondrial import may not be applicable to TAT-conjugated NDUFS8. The exact mechanism through which exogenous TAT fusion proteins are delivered into mitochondria remains unknown at this stage and further investigation is needed.

After the binding of TAT-conjugated proteins to cell membranes, the fusion proteins have to penetrate cell membranes to enter cells. Based on the available data, it appears that multiple cellular entry routes exist for TAT-derived cargoes, which include direct membrane translocation and various types of endocytotic processes [24]. Among them, macropinocytosis is the most recognized cell entry route for TAT-mediated protein transduction [36,37]. Following internalization by macropinocytosis, several reports indicated that TAT-conjugated cargoes have to escape from endosomes so that they can be released into the cytoplasm to execute the desired function [36,38]. On the basis of our current data of TAT-NDUFS8 from the transient transfection experiment and their comparison with the corresponding TAT-NDUFS8 transduction experiment (Figure 1 and Figure 3, respectively), the exogenously produced TAT-NDUFS8 from protein transduction appears to not be released from endosomes to the cytosol, as the fusion protein would otherwise not correctly enter mitochondria, just like the result shown in the transfection study. Therefore, we propose that the exogenously produced TAT-fused NDUFS8 may enter mitochondria via an as yet not well-known endosome-mitochondria juxtaposition route (contact or even fusion) exemplified by the entry of *Helicobacter pylori* vacuolating cytotoxin A (VacA) into mitochondria [39,40]. VacA is a pore-forming toxin of *H. pylori* and can enter host gastric cells via endocytosis. After internalization, this toxin is enclosed by endosomes. In one of the downstream pathways, the VacA-enriched endosomes can move close to mitochondria to form endosomes-mitochondria juxtaposition and further transport VacA into mitochondria to trigger apoptosis in host cells. Based on the discovered VacA traffic pathway, it would be interesting in the future to explore the possible involvement of endosomes-mitochondria juxtaposition in the import of TAT-conjugated NDUFS8 into mitochondria through protein transduction.

## 4. Materials and Methods

### 4.1. Molecular Cloning of NDUFS8 and Its TAT-Containing Constructs

A DNA fragment of *NDUFS8* with *Nde*I and *Xho*I restriction enzyme cutting sites was amplified by pfu polymerase with a primer pair, *Nde*I-NDUFS8-F and NDUFS8-*Xho*I-R, using the pcDNA4-*NDUFS8* construct as the template [41]. The pET28b plasmid with *TAT* sequence insert (provided by Dr. Steven F. Dowdy from the University of California, San Diego School of Medicine, CA, USA) was digested with *Nde*I and *Xho*I restriction enzymes and then ligated to the processed *NDUFS8* PCR product to generate the pET28b*-TAT-NDUFS8* construct. For the generation of the pET28a*-NDUFS8-TAT* construct, a DNA fragment of *NDUFS8* with *Eco*RI and *Xho*I restriction enzyme cutting sites was amplified by pfu polymerase with a primer pair *Eco*RI-NDUFS8-F and NDUFS8-*Xho*I-R using the pcDNA4-*NDUFS8* construct as the template. The processed PCR product was then ligated to the pET28a plasmid pre-digested with *Eco*RI and *Xho*I restriction enzymes to generate the pET28a-*NDUFS8* construct. Using the obtained pET28a-*NDUFS8* construct as the template, a primer with an extending oligonucleotide *TAT*-*Eco*RV was designed and applied to PCR amplification to generate the *Eco*RI-*NDUFS8*-*Xho*I-*TAT*-*Eco*RV insert. The sticky end of the *Xho*I restriction site generated by pre-digestion of the pET28a plasmid with *Eco*RI and *Xho*I restriction enzymes was filled to become a blunt end with T4 DNA polymerase. Finally, the *Eco*RI-*NDUFS8*-*Xho*I-*TAT*-*Eco*RV insert was ligated to the above modified plasmid to generate the pET28a-*NDUFS8-TAT* construct. The resulting pET28b*-TAT-NDUFS8* and pET28a*-NDUFS8-TAT*, and previously generated pET28a*-NDUFS8,* were used in later experiments for TAT-NDUFS8, NDUFS8-TAT, and NDUFS8 overexpression in *Escherichia coli* and protein purification, respectively. Furthermore, using the pET28b*-TAT-NDUFS8* construct as the template with primers *Hin*dIII-TAT-NDUFS8-F and NDUFS8-*Xho*I-R, a DNA fragment containing *TAT-NDUFS8* with *Hin*dIII and *Xho*I cutting sites was generated and ligated with the pcDNA4 vector to form the pcDNA4*-TAT-NDUFS8* construct. Similarly, using the pET28a*-NDUFS8-TAT* construct as the template with primers *Eco*RI-NDUFS8-2-F and NDUFS8-TAT-*Xba*I-R, a DNA fragment containing *NDUFS8-TAT* with *EcoRI* and *XbaI* cutting sites was generated and ligated with the pcDNA4 plasmid to form the pcDNA4*-NDUFS8-TAT* construct. The pcDNA4*-TAT-NDUFS8*, pcDNA4*-NDUFS8-TAT*, and previously generated pcDNA4*-NDUFS8* plasmids were used in later experiments for transient transfection. All oligonucleotide sequences of the primers used in this study are listed in Table 1.

### 4.2. Overexpression and Purification of TAT-NDUFS8, NDUFS8-TAT, and NDUFS8 Proteins

*E. coli* BL21 (DE3) cells transformed with pET28b-*TAT-NDUFS8*, pET28a-*NDUFS8-TAT*, or pET28a-*NDUFS8* were cultivated in Luria-Bertani medium overnight at 37 °C. The bacterial culture was then added into Yeast Extract Tryptone (2YT) medium and cultivated at 37 °C until the OD_600nm_ value reached 0.8. Then, 1.0 mM isopropy-β-D-thiogalactoside (IPTG; VWR-Amresco, Radnor, PA, USA) was used to induce protein overexpression for 5 h. Then, bacterial cells were collected by centrifugation with an R10A3 rotor at 5200× *g* rpm for 15 min (Hitachi, Tokyo, Japan) and resuspended in buffer Z (8 M urea, 100 mM NaCl and 20 mM 4-(2-hydroxyethyl)-1-piperazineethanesulfonic acid (HEPES), pH 8.0). After, sonication was used to rupture cells (conditions: 10 s on/off for 30 min). The supernatant and pellet fractions of cells were collected by centrifugation (Hitachi R15A rotor, 14,000× *g* rpm for 20 min at 4 °C). The collected TAT-NDUFS8, NDUFS8-TAT, and NDUFS8 supernatant fractions were added into the affinity chromatography column packed with IMAC Sepharose 6 FastFlow resins (GE Healthcare, Chalfont St. Giles, UK) and CoCl_2_ chelating reagents. The protein bound resins were sequentially washed with the elution buffer containing different concentrations of imidazole (20–1000 mM, pH 5.5). The fractions collected from 250 and 500 mM imidazole elution were concentrated using an Amicon Ultra concentrator (Merck-Millipore, Darmstadt, Germany) and exchanged with PBS. The purified proteins were further analyzed by sodium dodecyl sulfate-polyacrylamide gel electrophoresis (SDS-PAGE). Finally, 20% glycerol was added to the collected recombinant proteins and kept at −20 °C until later analyses.

### 4.3. Generation of an NDUFS8 Knockdown Stable Cell Line

A short-hairpin RNA (shRNA) clone targeting base pairs 243–263 of the NDUFS8 coding region was purchased from National RNAi Core Facility (NRC, Taipei, Taiwan). The short-hairpin oligonucleotide sequence (shRNA-C3) inserted in the plasmid pLKO.1-puro is shown in Table 1. Cells in 6-well culture plates were transfected with the shRNA-C3-containing plasmid by Lipofectamine 2000 transfection reagent (Thermo Fisher Scientific, Waltham, MA, USA). After 24 h incubation, cells were transferred to 10 cm culture dishes and selected in a medium containing 2.5 μg/mL puromycin (Sigma-Aldrich, St. Louis, MO, USA) for 4 weeks.

### 4.4. Cell Culture

T-REx-293 cells, human embryonic kidney cells with a tetracycline-regulated expression system (Thermo Fisher Scientific), and their derived NDUFS8 stable knockdown cells (shRNA-C3) were cultivated in Dulbecco’s modified eagle medium (DMEM) containing 10% fetal bovine serum (FBS; Biological Industries, Beit Haemek, Israel), 100 U/mL penicillin, and 100 μg/mL streptomycin (Biological Industries) with 5% CO_2_ at 37 °C. shRNA-C3 cells were maintained in the above cell culture medium containing 2.5 μg/mL puromycin (Sigma-Aldrich).

### 4.5. Transient Transfection

T-REx-293 cells were transfected with MaestroFectin transfection reagents (MaestroGen, Hsinchu City, Taiwan) as recommended by the manufacturer. Cells were seeded in 6 cm dishes and grown to 70–80% confluency before the transfection. The transfection procedure was conducted as follows: 3 μL MaestroFectin transfection reagent was first added to 90 μL serum-free medium (without antibiotics) in an Eppendorf tube and mixed gently. After incubating for 5 min at room temperature, 3 μg plasmid DNA was added to the diluted MaestroFectin transfection reagent and mixed gently. The mixture was incubated for 15 min at room temperature to form a MaestroFectin transfection reagent-DNA complex, and then 500 ng/mL tetracycline was added to the cells to induce the overexpression of target proteins for 18 h at 37 °C.

### 4.6. Transduction of Proteins into Cells

Cells were cultured in 6 or 10 cm dishes to 70–80% confluency. Before protein transduction, cells were washed once with PBS and then incubated with different amounts of TAT-NDUFS8, NDUFS8-TAT, or NDUFS8 in serum-free growth medium for 3–48 h. We added 10% FBS to the cell culture when the incubation time was more than 3 h to prevent cell starvation.

### 4.7. Subcellular Fractionation

The cytosolic and mitochondrial fractions of cells were separated by differential centrifugation with some modifications to a previously established method [39]. All the steps of centrifugation were performed at 4 °C. Briefly, cells transfected with expression plasmids or treated with transduction proteins were obtained by trypsinization. The resulting cells were resuspended with 3 times the pellet volume of homogenization buffer (HB; 250 mM sucrose, 3 mM imidazole, and 1mM ethylenediaminetetraacetic acid (EDTA); pH 7.4) on ice and centrifuged at 1300× *g* for 10 min. Cells were then resuspended with 4 times the pellet volume of HB and homogenized using 45 strokes with a glass homogenizer. The obtained homogenate was centrifuged at 3000× *g* for 10 min. The post-nuclear supernatant (PNS) was transferred to a clean tube for further centrifugation at 12,000× *g* for 10 min. The supernatant collected was the cytosolic fraction. The nuclear pellet (NP) was resuspended with 0.4 mL assay buffer (120 mM mannitol, 80 mM KCl, 1 mM EDTA, and 20 mM HEPES; pH 7.4) and centrifuged at 600× *g* for 10 min again. The resulting supernatant was layered onto a cushion of 1 M mannitol in the assay buffer (0.6 mL). After centrifugation at 9000× *g* for 15 min, the obtained pellet was resuspended in 0.5 mL assay buffer and further centrifuged at 10,000× *g* for 10 min. Finally, the pellet was resuspended in 30 μL lysis buffer as the mitochondrial fraction.

### 4.8. Trypsin Treatment

After subcellular fractionation, the mitochondrial pellet was incubated with trypsin to the concentration of 1 μg trypsin/100 μg mitochondrial pellet at 37 °C for 5 min to obtain the trypsin-treated mitochondrial fraction.

### 4.9. Carbonyl Cyanid p-(Trifluoromethoxy) Phenylhydrazone Treatment

Cells were first cultivated in the cell culture medium containing 10 μM carbonyl cyanid *p*-(trifluoromethoxy) phenylhydrazone (FCCP) for 18 h. Later, the serum-free medium containing 0.06 mg/mL TAT-NDUFS8 or NDUFS8-TAT was replaced by the original cell culture medium and incubated for another 3 h. The resulting cell culture was applied for subcellular fractionation.

### 4.10. Prepartion of Total Cell Lysates

Cells were first cultivated in 6 cm dishes to 90% confluency and then collected by trypsinization, followed by washing once with PBS. Later, the lysis buffer (150 mM NaCl, 5 mM EDTA, 1% triton-x 100, and 10 mM Tris-Cl; pH 7.4) was added to cells for cell disruption to obtain the total cell lysates.

### 4.11. Protein Electrophoresis and Immunoblotting Analysis

A bicinchoninic acid (BCA) protein assay kit (Thermo Fisher Scientific) with bovine serum albumin (BSA) as the standard was used to determine the protein concentration of samples. We added 4× protein sample dye into the samples and the mixtures were boiled for 10 min before gel loading. Protein samples were separated by 10% SDS-PAGE gels. The resolved gels were then transferred to 0.45 µm nitrocellulose membranes (GE Healthcare, Chicago, IL, USA) at a 400 mA current for 90 min. The transferred membranes were blocked with 5% skim milk in PBS for 1 h at room temperature, and then incubated at 4 °C overnight with one of the following primary antibodies: rabbit anti-His antibody (Bioman, New Taipei city, Taiwan; 1:5000 dilution), rabbit anti-NDUFS8 antibody (GeneTex, Hsinchu City, Taiwan; 1:1000 dilution), mouse anti-ATPase α subunit antibody (Thermo Fisher Scientific; 1:10,000 dilution), mouse anti-β tubulin antibody (Santa Cruz Biotechnology, Dallas, TX, USA; 1:500 dilution), mouse anti-GAPDH antibody (Merck-Millipore; 1:10,000 dilution), mouse anti-Tom20 antibody (Santa Cruz Biotechnology; 1:250 dilution), or mouse anti-NDUFS3 antibody (Thermo Fisher Scientific; 1:500 dilution). After 5 washes with PBST (PBS containing 0.02% Tween-20) for 7 min each, the membranes were incubated with the secondary antibodies of goat anti-mouse IRDye 680LT (LI-COR Biosciences, Lincoln, NE, USA; 1:10,000 dilution) or goat anti-rabbit IRDye 800CW (LI-COR Biosciences; 1:10,000 dilution) for 45 min in a light-proof container. The membranes were then washed 5 times for 7 min each with PBST and scanned using a LI-COR Odyssey^®^ infrared imaging system (LI-COR Biosciences).

### 4.12. Cell Viability Assay

The 3-(4,5-cimethylthiazol-2-yl)-2,5-diphenyl tetrazolium bromide (MTT) assay (Sigma-Aldrich, St. Louis, MO, USA) was used to determine the cell viability. A total of 5 × 10^4^ cells were seeded and cultivated in each well of 96-well plates for 24 h, followed by treatment with different amounts of TAT-NDUFS8 or NDUFS8-TAT recombinant protein for 48 h. Later, we added 250 μg/mL MTT to the cell culture for 2 h. The resulting yellowish solution was reduced to purple formazan in living cells, and 200 μL dimethyl sulfoxide (DMSO; Scharlau, Sentmenat, Spain) was then added to each well to dissolve the insoluble MTT product into a purplish solution. The cell viability index was measured at 565 nm by a Wallac 1420 VICTOR^3^ V plate reader (PerkinElmer, Waltham, MA, USA).

### 4.13. High-Resolution Clear Native Gel Electrophoresis (HrCNE) and Complex I In-Gel Activity Assay

T-REx-293 cells and the derived NDUFS8 stable knockdown cells (shRNA-C3) were cultured with or without 0.03 mg/mL TAT-NDUFS8 recombinant protein for different periods of time and then harvested by trypsinization. After PBS washing and centrifugation, cells were resuspended in ice-cold mitochondrial extraction buffer (250 mM sucrose, 10 mM KCl, 1.5 mM MgCl_2_, 1 mM EDTA, 20 mM HEPES/KOH, 1 mM dithiothreitol (DTT), 1 mM phenylmethylsulfonyl fluoride (PMSF), and protease inhibitor cocktail; Roche, Penzberg, Germany) and homogenized by 45 strokes with a glass homogenizer on ice. The homogenized suspensions were first centrifuged at 700× *g* for 15 min to remove cell debris, followed by centrifugation at 10,000× *g* for 10 min to obtain crude mitochondrial pellets. The pellets were weighed and solubilized on ice with 35 μL solubilization buffer (50 mM NaCl, 50 mM imidazole/ HCl, 2 mM 6-aminocaproic acid, and 1mM EDTA) per 20 mg of pellets. Later, 6 μL 20% (*w*/*v*) *n*-dodecyl β-D-maltoside (DDM; Sigma-Aldrich) per 20 mg of pellets was added and mixed by flicking the Eppendorf tube. The resulting suspension was incubated on ice for 10 min, followed by centrifugation at 20,000× *g* for 20 min. The supernatant containing the DDM-solubilized mitochondrial complexes was collected and mixed with the loading buffer containing 10% glycerol and 0.1% Ponceus S. We used 6.5% acrylamide non-gradient native gel with anode buffer (25 mM imidazole/HCl, pH 7.0) and cathode buffer (50 mM Tricine, 7.5 mM imidazole/HCl, 0.05% deoxycholate, and 0.02% DDM, pH 7.0) to separate the mitochondrial protein complexes. The gel was first run at a voltage of 45 V with a fixed current of 20 mA for 30 min, followed by at a voltage of 100 V with a fixed current of 20 mA for 20 min, and then at a voltage of 250 V with a fixed current of 15 mA for 90 min at 4 °C. After the completion of HrCNE, the resulting native gels were applied in the following 3 assays: First, the protein profile in the native gel was observed by gel staining with Coomassie blue solution (0.1% Coomassie brilliant blue R-250 (Sigma-Aldrich), 50% methanol, and 10% glacial acetic acid) for 1 h and destained with the destaining solution (40% methanol and 10% glacial acetic acid) overnight. Secondly, the degree of NDUFS8 assembled in the mitochondrial complex I was detected using an immunoblotting analysis with rabbit anti-NDUFS8 antibody (GeneTex; 1:1000 dilution). The obtained signals were calculated by ImageJ software (NIH, Bethesda, MA, USA). The intensity of NDUFS8 in the samples was normalized by the loading control, complex V (detected by anti-ATPase α subunit antibody), at 100%. Lastly, the activity of complex I was assessed using the in-gel activity assay. The native gel was incubated with 5 mM Tris/HCl buffer (pH 7.4) containing 0.1 mg/mL NADH and 2.5 mg/mL nitroblue tetrazolium (NBT; USB, Cleveland, OH, USA) at room temperature for 30 min and then fixed with the fixing solution (50% methanol and 10% acetic acid) at room temperature for 30 min, followed by washing with ddH_2_O and scanned to quantify complex I in-gel activity.

### 4.14. Oxygen Consumption Measurement

Cells were cultured with or without the treatment of TAT-NDUFS8 (0.03 mg/mL) for 48 h and then harvested by trypsinization. After PBS washing and centrifugation, 5 × 10^6^ cells were resuspended in 1 mL of the respiration buffer (RB; 0.3 M mannitol, 10 mM KCl, 5 mM MgCl_2_, and 10 mM K_2_PO_4_; pH 7.4) and permeabilized by the addition of non-ionic detergent digitonin (Sigma-Aldrich, 60 μg/mL) under continuous shaking in a 15 mL tube for 2 min at room temperature. Then, 5 mL RB supplemented with 1 mg/mL BSA was added to the cell suspension to stop the membrane permeabilization. The resulting mixtures were then centrifuged at 1000× *g* for 5 min, and the obtained pellets were resuspended in 500 μL RB buffer supplemented with 1 mg/mL BSA and 0.5 mM ADP. The digitonin-permeabilized cell suspension was ready for oxygen consumption measurements. The assay was conducted by the Mitocell S200 micro respirometry system (Strathkelvin Instruments, North Lanarkshire, U.K.) after the addition of complex I substrates, 8 mM pyruvate, and 0.2 mM malate. The reaction was stopped by the addition of a complex I inhibitor, rotenone (3 μM).

### 4.15. Immunofluorescence Detection

Cells were seeded onto cover glasses in 24-well plates and grown to 70–80% confluency, followed by treatment with 0.03 mg/mL TAT-NDUFS8 for 3 h. To label mitochondria, 0.1 mM Mitotracker Red (Thermo Fisher Scientific) was were added to cells, which were cultivated for another 30 min. Cells were then fixed by adding the acetone/methanol mixture (acetone: methanol = 3:1 in volume proportion) for 5 min on ice. After that, cells were incubated with the culture medium for another 1 h at room temperature, followed by treatment with rabbit anti-His antibody (Bioman, New Taipei city, Taiwan; 1:100 dilution). After incubating at room temperature for 1 h, the cover glasses were washed with PBS 3 times for 5 min each. Then, cells were incubated with anti-rabbit IgG-FITC antibody (AnaSpec, Fremont, CA, USA; 1:100 dilution) at room temperature for 1 h, and washed with PBS 3 times for 5 min each. Lastly, the cover glasses were mounted with VECTASHIELD Mounting Medium (Vector Laboratories, Burlingame, CA, USA). Fluorescence was visualized using an LSM 5 PASCAL confocal microscope (ZEISS, Welwyn Garden City, Germany).

### 4.16. Statistical Analysis

Results are presented as mean and standard deviation (±SD) from n ≥ 3 independent experiments. Data were analyzed using the unpaired Student’s *t*-test with two-tailed distribution, and the results were considered significant when the *p* value was smaller than 0.05, 0.01, and 0.001.

## Figures and Tables

**Table 1:** The sequences of primers used in this study.

Name	Oligonucleotide Sequence (5′–3′) ^1,2^	Annotation
*Nde*I-NDUFS8-F	CGACATATGCGCTGCCTGACCACG	Designed for pET28b-*TAT-NDUFS8* with *Nde*I site
NDUFS8-*Xho*I-R	AGGCTGACTACTTGTATCGGCTCGAGATA	Designed for pET28b-*TAT-NDUFS8* with *Xho*I site
*Eco*RI-NDUFS8-F	GAGAATTCATGCGCTGCCTGACCACG	Designed for pET28a-*NDUFS8* with *Eco*RI site
NDUFS8-*Xho*I-R	AGGCTGACTACTTGTATCGGCTCGAGATA	Designed for pET28a-*NDUFS8* with *Xho*I site
*Eco*RI-NDUFS8-2-F	CTTTAAGAAGCAGATATACTTTAAGAACGAGATATAGAATTCATGCGCTGCCTG	Designed for pET28a-*NDUFS8-TAT* with *Eco*RI site
NDUFS8-*Eco*RV-R	ATTGATATCGCCTCTTCGTCGCTGTCTCCGCTTCTTCCTCTCGAGCCGATACAAGTA	Designed for pET28a-*NDUFS8-TAT* with *Eco*RV site
*Hin*dIII-TAT-NDUFS8-F	GACAAGCTTATGGGCAGGAAGAAGCGGA	Designed for pcDNA4-*TAT-NDUFS8* with *Hin*dIII site
NDUFS8-*Xho*I-R	AGGCTGACTACTTGTATCGGCTCGAGATA	Designed for pcDNA4-*TAT-NDUFS8* with *Xho*I site
*Eco*RI-NDUFS8-2-F	CTTTAAGAAGCAGATATACTTTAAGAACGAGATATAGAATTCATGCGCTGCCTG	Designed for pcDNA4-*NDUFS8-TAT* with *Eco*RI site
NDUFS8-TAT-*Xba*I-R	ATTTCTAGAGCCTCTTCGTCGCTGTCTCCGCTTCTTCCTCTCGAGCCGATACAAGTA	Designed for pcDNA4-*NDUFS8-TAT* with *Xba*I site
shRNA-C3	CCGG*CATCAACTACCCGTTCGAGAA*CTCGAG*TTCTCGAACGGGTAGTTGATG*TTTTTG	Designed for the knokdown of *NDUFS8* (target sequence: base pairs 243–263)

^1^ The sequences of the restriction enzyme cutting sites are underlined. ^2^ The sequences of the target sites for NDUFS8 knockdown are italicized.

## Data Availability

Data sharing is not applicable to this article as no new data were created or analyzed in this study.
